# MicroRNA-129 modulates neuronal migration by targeting *Fmr1* in the developing mouse cortex

**DOI:** 10.1038/s41419-019-1517-1

**Published:** 2019-03-25

**Authors:** Chao Wu, Xiaoling Zhang, Pan Chen, Xiangbin Ruan, Wei Liu, Yanchao Li, Changjie Sun, Lin Hou, Bin Yin, Boqin Qiang, Pengcheng Shu, Xiaozhong Peng

**Affiliations:** 1 The State Key Laboratory of Medical Molecular Biology, Neuroscience Center, Medical Primates Research Center and Department of Molecular Biology and Biochemistry, Institute of Basic Medical Sciences, Chinese Academy of Medical Sciences, School of Basic Medicine Peking Union Medical College, 100005 Beijing, China; 2 Institute of Medical Biology, Chinese Academy of Medical Science and Peking Union Medical College, 650118 Kunming, China

**Keywords:** Neurodevelopmental disorders, Neuronal development

## Abstract

During cortical development, neuronal migration is one of the most important steps for normal cortical formation and function, and defects in this process cause many brain diseases. However, the molecular mechanisms underlying this process remain largely unknown. In this study, we found that miR-129-5p and miR-129-3p were expressed in both neural progenitor cells and cortical neurons in the developing murine cortex. Moreover, abnormal miR-129 expression could block radial migration of both the deeper layer and upper layer neurons, and impair the multipolar to bipolar transition. However, antagomir-mediated inhibition resulted in overmigration of neurons. In addition, we showed that Fragile X Mental Retardation gene 1 (*Fmr1*), which is mutated in the autism spectrum disorder fragile X syndrome, is an important regulatory target for miR-129-5p. Furthermore, *Fmr1* loss-of-function and gain-of-function experiments showed opposite effects on miR-129 regulation of neuronal migration, and restoring *Fmr1* expression could counteract the deleterious effect of miR-129 on neuronal migration. Taken together, our results suggest that miR-129-5p could modulate the expression of fragile X mental retardation 1 protein (FMRP) to ensure normal neuron positioning in the developing cerebral cortex.

## Introduction

The mammalian neocortex is highly organised into a six-layered structure and involved in a variety of higher cognitive, sensory, emotional, and motor functions. During the development of the neocortex, a remarkably diverse array of excitatory projection neuron types arises primarily from progenitors in the two germinal zones (GZs) of the dorsal telencephalon: the ventricular zone (VZ) and the subventricular zone (SVZ)^1,2^. Radial glial cells (RGCs) in the VZ can produce neurons directly by asymmetric divisions that generate one RGC and one neuron, and can also indirectly generate amplified intermediate progenitors (IPs) in the SVZ^3–9^. Neurogenesis and layer formation are precisely orchestrated so that early-born neurons occupy the deep layers (layer VI, then layer V) and later-generated cortical plate cells migrate past older neurons and settle in progressively more superficial layers (layer IV, then layer II/III), resulting in a “inside-out” structure^2,10–14^. Disruption of the migration process has been associated with various pathologies, such as lissencephaly, heterotopia, pachygyria, schizencephaly and autism spectrum disorders (ASD)^15–17^. However, the molecular mechanisms underlying this process have not been fully elucidated.

MicroRNAs (miRNAs) are endogenous ~22 nucleotide noncoding RNAs that can specifically bind to the 3′ untranslated region (3′UTR) of target mRNAs and mediate their degradation or translation inhibition^18^. Although many miRNAs have been shown to be involved in regulating neuronal stem cell proliferation and differentiation^19–25^, whether miRNAs are involved in neuronal migration remains unknown. However, a few clues have been reported. MiR-9 and miR-132-dependent repression of Foxp2 was implicated in the radial migration^26^. MiR-379-410 cluster miRNAs were reported to regulate neuronal migration by fine-tuning N-cadherin^27^. MiR-22 and miR-124 promoted radial migration by targeting CoREST^28^. Recently, miR-128 was shown to regulate neuronal migration by binding to the 3′UTR of Phf6^29^.

During our previous work using genome-wide microarray screens to find factors regulating cortical neuron fate determination, we identified miR-129, a conserved miRNA in humans, mice, rats and zebrafish, which had a crucial role in cortical migration. The mouse genome contains two miR-129 genes: miR-129-1 (Gene ID: 387237) located on chromosome 6qA3.3 and miR-129-2 (Gene ID: 723953) located on chromosome 2pE1. MiR-129 was reported as a tumour suppressor with decreased expression in various types of human cancers, which could regulate many cancer-related phenotypes, such as DNA methylation, cell proliferation, apoptosis, cell cycle, and metastasis^30–35^. During the retinal development, miR-129 was shown to inhibit Xotx2 and Xvsx1 to block the generation of bipolar neurons^36^. FOXP2, which is associated with cortical development, has been identified as a target gene of miR-129-5p^37^. MiR-129-5p is highly expressed at the synapse and regulates the metabolism of FMR1 mRNA^38^. Recently, functional crosstalk between miR-129-5p and Rbfox1 was shown to control neural network homoeostasis and epileptogenesis^39^. MiR-129-5p was also reported to be associated with Alzheimer’s disease (AD)^40^. However, its role in cortical development has not been characterised.

In this study, we have found that both miR-129-5p and miR-129-3p are expressed in neural progenitor cells (NPCs) and cortical neurons during cortical neurogenesis. Gain- and loss-of-function of miR-129 in neocortical progenitors disrupts the radial migration and impairs the multipolar to bipolar transition process. In addition, we identified *Fmr1* as a significant regulatory target for miR-129-5p, which is also involved in regulating RGCs and neuronal migration^41,42^. Coexpression of FMRP could rescue the physiological characteristics of the miR-129 gain-of-function phenotypes in both deeper layer (DL) and upper layer (UL) neurons. We have thus identified a novel miR-129-5p-*Fmr1* regulatory pathway that controls neuronal migration in the developing neocortex.

## Results

### Overexpression of miR-129 in neocortical progenitors perturbs neuronal migration

**Fig. 1:**
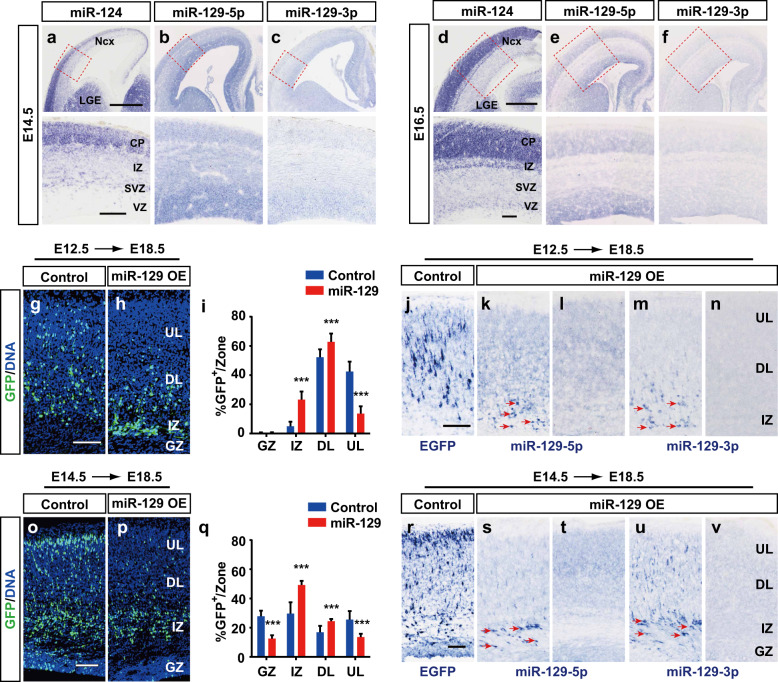
MiR-129 overexpression blocks neuronal migration. **a**–**f** The expression patterns of miR-124 (**a**, **d**), miR-129-5p (**b**, **e**), and miR-129-3p (**c**, **f**) were detected by in situ hybridization of coronal sections of E14.5 and E16.5 embryonic telencephalon. The images at the bottom of each panel are the higher magnification of the red dashed box in **a**–**f**. The scale bar is 500 μm in the top images of **a** and **c** with 100 μm scale bar in their bottom images. **g**–**i**, **o**–**q** Plasmids expressing GFP alone (control) and that coexpressing miR-129 were electroporated into the embryonic mouse brain at E12.5 or E14.5, and GFP-positive cells were quantified from the sections of E18.5 brains from different littermates (E12.5–E18.5, control: *n* = 3, miR-129: *n* = 6; E14.5–E18.5, control: *n* = 3, miR-129: *n* = 3). The results are expressed as the mean ± SD. Comparisons were performed by Student’s *t*-test, and the statistically significant *P*-values are shown as *P < 0.05, **P < 0.01, and ***P < 0.001. The scale bar is 100 μm in **g** and **o**. **j**–**n**, **r**–**v** Overexpressed miR-129-5p and miR-129-3p in the E12.5–E18.5 (**k**, **m**) and E14.5–E18.5 (**s**, **u**) electroporated samples were examined using LNA probes specific to miR-129-5p and miR-129-3p. EGFP probe (**j**, **r**) and the contralateral untransfected side (**l**, **n**, **t**, **v**) were used as controls. Red arrows indicate the overexpression signals. The scale bar is 100 μm in **j** and **r**. GZ germinal zone, IZ intermediate zone, DL deep layer, UL upper layer

To better understand the function of miR-129 during neocortical development, we first examined its expression in the embryonic dorsal forebrain. We performed *in situ* hybridization (ISH) experiments with miRNA probes modified by locked nucleic acid (LNA)^43^ complementary to the mature miRNA to ascertain their expression patterns in the developing neocortex at E14.5 and E16.5. We used miR-124, a neuron-specific miRNA, as a positive control^24^. Consistent with an earlier report, miR-124 was expressed in both the mature neurons in the cortical plate (CP) and the migrating neurons in the SVZ and IZ but not in the progenitor cells in the VZ (Fig. 1a, d). MiR-129-5p was highly expressed in the dorsal telencephalic progenitor cells in the VZ and mature neurons in the CP (Fig. 1b, e). Expression of this miRNA decreased from E14.5 to E16.5 in the neural progenitor cells (Fig. 1b–e). In contrast, miR-129-3p expression was much lower than miR-129-5p (Fig. 1c, f). We noted that most neurons in the CP are derived from NPCs in the VZ; thus, miR-129-5p and miR-129-3p are expressed primarily in neocortical VZ progenitors and maintain their expression in the post-mitotic neurons.

To gain insight into the possible role miR-129 has in neocortical development, we amplified *pre-miR-129-2* with its flanking sequence and cloned it into the pCIG vector, which allows miR-129 to be highly expressed in vivo. We performed in utero electroporation (IUE) experiments to introduce miR-129 overexpression (OE) and control plasmid into proliferating cells at E12.5 and E14.5. We collected the embryos at E18.5 and assessed the positions of GFP-expressing (GFP^+^) electroporated cells in the neocortices. In the E12.5 electroporated embryos, the GFP^+^ cells were distributed both in the deeper and upper neocortical layers in the control (Fig. 1g, i). In contrast, there was a striking migratory block in miR-129 OE embryos, and miR-129 OE cells accumulated in the IZ (23.2 ± 5.5% vs. 5.0 ± 3.2%) instead of migrating into the CP (Fig. 1h, i). When the plasmid was transfected at E14.5, some of the electroporated cells already migrated to the top layer of CP, and many GFP^+^ cells remained during migration or in the GZ (Fig. 1o, q). Overexpression of miR-129 also caused aberrant neuronal migration, and many more transfected cells were distributed in the IZ (49.3 ± 9.5% vs. 29.7 ± 7.7%), while fewer cells were observed in the UL and GZ (Fig. 1p, q). Moreover, we used ISH to observe the expression of the exogenous miR-129 in the transfected neocortices and the EGFP probe to detect the transfected cells in the control embryos (Fig. 1j, k, m, r, s and u). We observed clear overexpression signals in the transfected side of the cerebral cortex compared to the contralateral untransfected side (Fig. 1l, n, t and v). In the E18.5 neocortex, miR-129 OE cells with strong signals for miR-129-5p and miR-129-3p were mainly distributed in the IZ, similar to the distribution patterns of GFP^+^ cells (Fig. 1h, k, m, p, s and u). Thus, these results suggest that miR-129 could regulate the radial migration of both the deep layer and upper layer neurons.

### Abnormal miR-129 expression leads to the reduction of intermediate neural progenitors

**Fig. 2:**
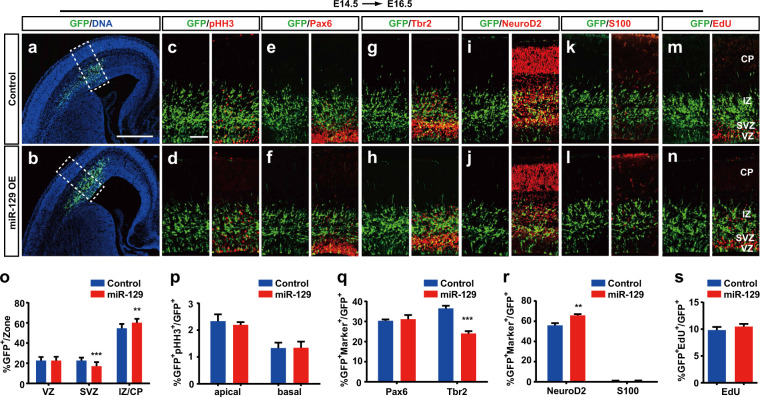
Ectopic expression of miR-129 reduces intermediate neural progenitors. **a**–**n** MiR-129 expression plasmids and control plasmids were electroporated into embryonic brain at E14.5, and brain sections of E16.5 were stained with pHH3, Pax6, Tbr2, NeuroD2 and S100 antibodies or using an imaging kit for EdU staining. The scale bar is 500 μm in **a** and 100 μm in **c**. **o** Quantification of GFP^+^ cells in different regions from different littermates (control: *n* = 3, miR-129: *n* = 4). **p**–**s** Quantification of GFP^+^ cells coexpressing the indicated markers from different littermates (control: *n* = 3, miR-129: *n* = 4). The results are expressed as the mean ± SD. Comparisons were performed by Student’s *t*-test, and the statistically significant *P*-values are shown as *P < 0.05, **P < 0.01 and ***P < 0.001. VZ ventricular zone, SVZ subventricular zone, IZ intermediate zone, CP cortical plate

MiR-129 is highly expressed in neocortical progenitors, and miR-129 OE could affect the distribution of cortical neurons generated from GZ. Therefore, we further dissected the role of miR-129 in the fate determination of NPCs. To test whether miR-129 could perturb the RGC to IP transition, we performed E14.5–E16.5 electroporation. After 48 h of electroporation, in the control embryos, most of the GFP^+^ cells (54.7 ± 4.3%) were distributed in the IZ, 22.6 ± 3.6% and 22.7 ± 2.9% of the transfected cells were positioned in the VZ and SVZ, respectively (Fig. 2a, o). In contrast, fewer miR-129-transfected cells were observed in the SVZ (17.1 ± 4.1%), leading to an increase in the number of GFP^+^ cells in the IZ (60.3 ± 3.8%) (Fig. 2b, o). Thus, these results suggest that miR-129 overexpression did not affect the RGC to IP transition but caused a reduction in IPs.

Interkinetic nuclear migration (INM) is a hallmark of vertebrate neural progenitors, and the position of stem cells is changed dynamically during the cell cycle. To test whether miR-129 could affect INM, we stained the neocortex for pHH3 (Fig. 2c, d), which is an M-phase marker. We found that there was no difference between the control and miR-129 overexpressing neocortex (Fig. 2p), suggesting that miR-129 has no influence on the apical-to-basal mitotic transition.

To further detect the function of miR-129 in NPC fate determination, we first examined the markers for RGCs and IPs, Pax6 and Tbr2, respectively^4^. At 48 h after E14.5 electroporation, the proportion of miR-129 OE cells coexpressing Pax6 was similar to that of the control-transfected cells (Fig. 2e, f and q). However, there was a significant decrease in the miR-129 OE cells coexpressing Tbr2 (Fig. 2g, h and q). We also examined GFP^+^ cell fate by co-staining with the neuron marker NeuroD2 and the astrocyte marker S100 and found that more GFP^+^NeuroD2^+^ cells were observed during miR-129 overexpression (Fig. 2i, j and r), and almost no GFP^+^ S100^+^ cells were detected in the E16.5 mouse neocortex (Fig. 2k, l and r). Thus, these results suggest that miR-129 could promote neuronal differentiation.

Next, we wanted to determine whether miR-129 could regulate the proliferation of NPCs. The thymidine analogue 5-ethynyl-2′-deoxyuridine (EdU) was injected into the electroporated mice 30 minutes before killing. Compared to the control neocortices, miR-129 overexpression did not alter the percentage of GFP^+^EdU^+^/GFP^+^ proliferating S-phase progenitors (Fig. 2m, n and s). Our results demonstrate that miR-129 does not affect the proliferative capacity of RGCs but promote IPs differentiation.

### MiR-129 overexpression does not alter the laminar fate of deep and upper layer neurons

**Fig. 3:**
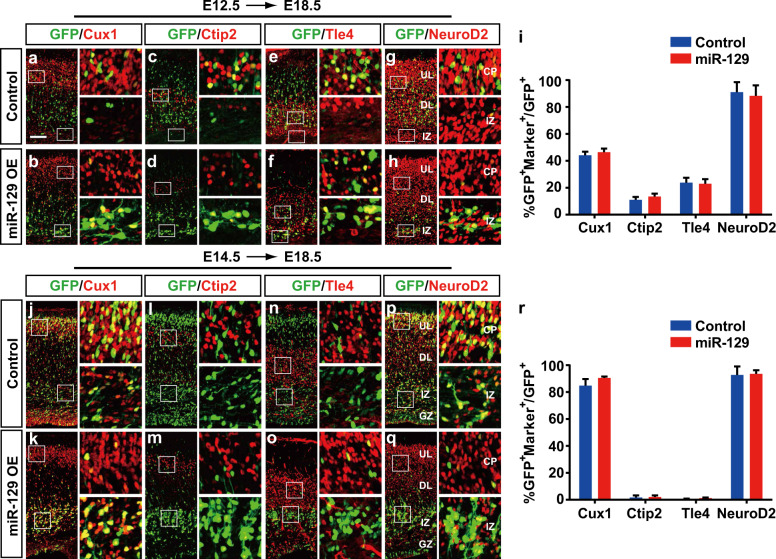
MiR-129 overexpression does not affect laminar fates of cortical neurons. **a**–**h** and **j**–**q** MiR-129 expression plasmids and control plasmids were electroporated into embryonic brains at E12.5 (**a**–**h**) or E14.5 (**j**–**q**), and brain sections of E18.5 embryos were stained with Cux1 (**a**, **b**, **j**, **k**), Ctip2 (**c**, **d**, **l**, **m**), Tle4 (**e**, **f**, **n**, **o**) or NeuroD2 (**g**, **h**, **p**, **q**) antibodies. Images next to each panel are high-magnification pictures of boxed regions in the CP and IZ to show the clear colocalization of GFP^+^ cells with different layer markers. The scale bar is 100 μm in **a**. **i**, **r** GFP^+^ cells coexpressing the indicated markers were quantified from different littermates of E12.5–E18.5 electroporated samples (control: *n* = 3, miR-129: *n* = 6) and E14.5–E18.5 electroporated samples (control: *n* = 3, miR-129: *n* = 3). For Ctip2 coexpressing cells, only the cells in the layer V were counted. GZ germinal zone, IZ intermediate zone, DL deep layer, UL upper layer. The results are expressed as the mean ± SD. Comparisons were performed by Student’s *t*-test, and the statistically significant *P* values are shown as *P < 0.05, **P < 0.01 and ***P < 0.001

To gain insight into the mechanism of the migration defect caused by miR-129, we tested whether ectopic miR-129 expression indirectly affects migration by interfering with the specification of cortical neuron identity. We introduced either the plasmid expressing miR-129 or the GFP control to the dorsal forebrain at E12.5 and E14.5 and analysed the phenotypes at E18.5 (Fig. 3). We first examined the effect of miR-129 on deep layer neurons. After electroporation for 6 days, we stained the neocortices with Cux1, a marker for layer II–IV neurons; Ctip2, a marker highly expressed in layer V neurons; Tle4, a marker for neurons in layer VI; and NeuroD2, a pan-neuron marker. As expected, miR-129 caused severe migration defects, but miR-129-transfected cells coexpressing Cux1 (Fig. 3a, b), Ctip2 (Fig. 3c, d), Tle4 (Fig. 3e, f) and NeuroD2 (Fig. 3g, h) showed no changes compared to the control (Fig. 3i).

We further examined the influence of miR-129 on upper layer neuron identity and found most of them coexpressed Cux1 and NeuroD2, with a few coexpressing Ctip2 and Tle4 (Fig. 3j, l, n, p and r). Although, in the miR-129 OE neocortices, most of the GFP^+^ cells resided in the IZ (Fig. 3k, m, o and q), their cell identities showed no changes compared to the control (Fig. 3r). These results suggest that miR-129 overexpression does not regulate the laminar fate of the cortical neurons.

### MiR-129 regulates the multipolar to bipolar transition affecting neuronal migration

We next invested why ectopic expression of miR-129 could cause aberrant radial migration. Most newly born neurons will first undergo a series of morphological changes during multipolar migration in the IZ, and then they must transform to bipolar morphology before they start to migrate to the CP; perturbation of this process can block radial migration^8,44^. To determine whether abnormal miR-129 expression influences the morphology of migrating neurons, we dissected transfected neurons after E14.5–E16.5 electroporation (Fig. S1a–d). In the control and miR-129 OE electroporation experiments, the migrating neurons had both multipolar and bipolar neuronal morphologies. Most of the neurons with bipolar morphologies extended their processes towards the basal surfaces in the control (Fig. S1a, b), but significantly more miR-129-transfected neurons exhibited multipolar morphologies, even the bipolar neurons with misdirection of their leading processes (Fig. S1c, d). Therefore, abnormal miR-129 expression perturbs the multipolar to bipolar morphology transition of migrating cortical neurons.

### Inhibition of miR-129 leads to overmigration of upper layer neurons

**Fig. 4:**
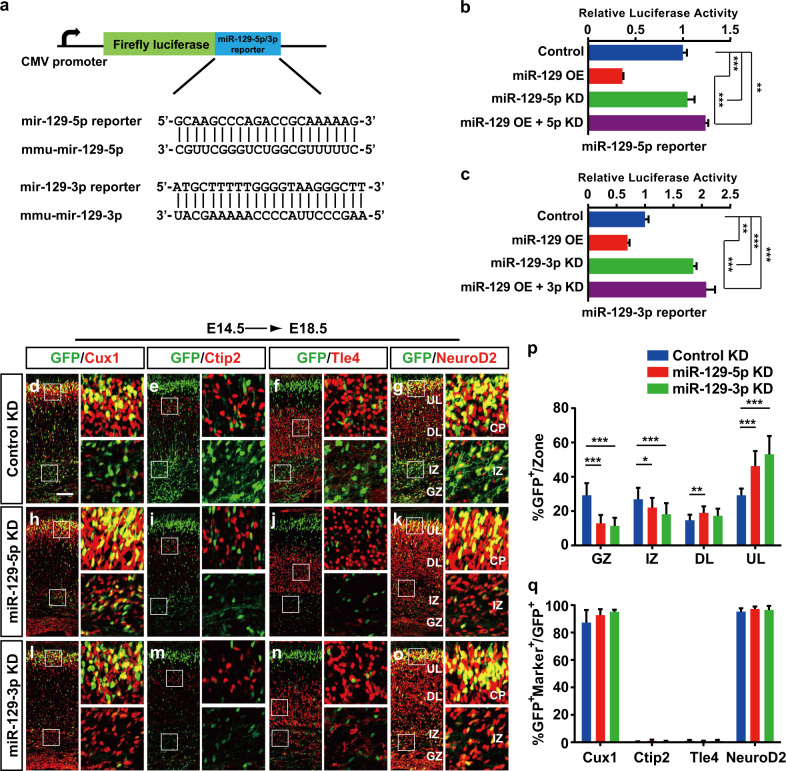
Knockdown of miR-129-5p or miR-129-3p resulted in excessive migration but did not affect neuronal fate determination. **a** A schematic diagram showing miR-129-5p reporter and miR-129-3p reporter plasmids containing sequences complementary to mature mouse miR-129-5p and miR-129-3p, respectively. **b**, **c** Dual-luciferase reporter assays were performed and the 293ET cells were co-transfected with miR-129-5p reporter or miR-129-3p reporter with the control and pCIG-miR-129 OE vector and miR-129-5p antagomir (KD) or miR-129-3p antagomir (KD); the cells were harvested after 48 h. **d**–**o** MiR-129-5p antagomirs (miR-129-5p KD) or miR-129-3p antagomirs (miR-129-3p KD) or control antagomirs (Control KD) were electroporated into embryonic brains at E14.5, and brain sections of E18.5 embryos were stained with Cux1 (**d**, **h**, **l**), Ctip2 (**e**, **i**, **m**), Tle4 (**f**, **j**, **n**) or NeuroD2 (**g**, **k**, **o**) antibodies. Images next to each panel are high-magnification pictures of boxed regions in the CP and IZ to show the clear colocalization of GFP^+^ cells with different layer markers. The scale bar is 100 μm in **d**. **p**, **q** Quantification of the GFP^+^ cell distribution and coexpression with the indicated markers in the control KD (*n* = 5), miR-129-5p KD (*n* = 4) and miR-129-3p KD (*n* = 4) embryos. For Ctip2 coexpressing cells, only the cells in layer V were counted. GZ germinal zone, IZ intermediate zone, DL deep layer, UL upper layer. The results are expressed as the mean ± SD. Comparisons were performed by Student’s *t*-test, and the statistically significant *P*-values are shown as *P < 0.05, ** *P* < 0.01 and ***P < 0.001

To further determine whether the loss of miR-129 expression also influences neocortical development, we used stable miRNA antagomirs to knockdown miR-129-5p and miR-129-3p in vivo. We performed luciferase assays to validate the effectiveness of miR-129-5p and miR-129-3p antagomirs in 293ET cells using their target reporters, which contained perfectly complementary sequences to miR-129-5p or miR-129-3p (Fig. 4a). As shown, both the miR-129-5p antagomir and miR-129-3p antagomir could significantly block the miR-129 inhibition of the reporters and cause an obvious increase in luciferase activity (Fig. 4b, c).

We then introduce the miR-129-5p or miR-129-3p antagomirs into the mouse dorsal telencephalon and examined their effects on neocortical development. At E18.5, the distribution of GFP^+^ cells in miR-129-5p or miR-129-3p knockdown (KD) neocortices was distinct from that of the control, with a clear shift in neuronal position towards the top of the neocortical (Fig. 4d–p), causing an “overmigration” phenotype. We further examined whether miR-129-5p or miR-129-3p KD could affect the laminar fate of transfected neurons. We stained the electroporated samples with Cux1, Ctip2, Tle4 and NeuroD2 and found that miR-129-5p KD did not alter the percentage of GFP^+^ cells coexpressing with Ctip2 and Tle4 (Fig. 4e, f, i, j and q), and the differentiated cells distributed outside of the GZ also showed no changes in coexpression of Cux1 and NeuroD2 (Fig. 4d, g, h, k and q). And there was also no significant increase in coexpression of Cux1, NeuroD2, Ctip2 and Tle4 cells in miR-129-3p KD neocortices compared to littermate controls (Fig. 4l–o and q). Given the phenotypes observed above, we concluded that miR-129 is necessary for the normal migration of cortical neurons during corticogenesis.

### The Fragile X syndrome gene, *Fmr1*, is a direct target of miR-129-5p during neuronal migration

To identify regulatory target genes for miR-129-5p and miR-129-3p that might be responsible for the aberrant migration, we first used prediction algorithms including TargetScan and PicTar to search for potential target genes. We selected 21 candidate genes with known or suspected roles in neuronal migration for further studies. We used dual-luciferase reporter assays to confirm whether these genes are regulated by miR-129-5p and miR-129-3p (data not shown). We concentrated on the Fragile X syndrome gene *Fmr1* because mutation of this gene was the major cause of this neurodevelopmental disorder, which was associated with aberrant neuronal migration. *Fmr1* was also reported to regulate neuronal migration in the developing mouse brain^41^. Another study demonstrated that FMR1 was a direct target gene of miR-129-5p in humans^38^.

To compare the *Fmr1* mRNA expression patterns, we used a published database of single-cell transcriptomics in the mouse neocortex^45^ and performed ISH to ascertain the *Fmr1* expression patterns at E14.5 and E16.5 (Fig. S2). *Fmr1* was detected throughout the cortex at E14.5 and E16.5 in the developing mouse neocortex, consistent with the single-cell transcriptomics (Fig. S2a, b and c).

**Fig. 5:**
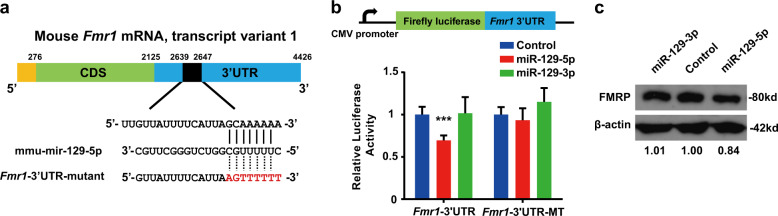
*Fmr1* is a direct target of miR-129-5p. **a** A schematic diagram showing a miR-129-5p binding site in the *Fmr1* 3′UTR. The mutated seed sequence is in red. **b** Luciferase reporter containing wild type or mutated 3′UTR of *Fmr1* was co-transfected with miR-129-5p, miR-129-3p or control mimics and the normalised luciferase activity was determined. The results are expressed as the mean ± SD. Comparisons were performed by Student’s *t*-test, and the statistically significant *P*-values are shown as *P < 0.05, **P < 0.01 and ***P < 0.001. **c** FMRP protein levels in miR-129-5p, miR-129-3p or negative control (NC) mimics-transfected N1E-115 cells were detected by western blot analysis using a specific FMRP antibody. β-actin was used as the loading control

There is one potential, conserved binding site for miR-129-5p in the mouse *Fmr1* mRNA (Fig. 5a). We used a dual-luciferase reporter assay to confirm whether *Fmr1* is also a target gene of miR-129-5p in mice. As expected, the luciferase activity was strongly reduced in synthetic miR-129-5p mimics-transfected 293ET cells (Fig. 5b). When the complementary seed sequence in the 3′UTR of *Fmr1* mRNA was mutated (Fig. 5a), the reduction of luciferase activity caused by miR-129-5p overexpression was abolished, indicating the specificity of miR-129-5p for *Fmr1* (Fig. 5b). In contrast, overexpression of miR-129-3p did not affect luciferase activity in the cells transfected with normal and mutant 3′UTRs of *Fmr1* compared to the control (Fig. 5b).

To determine whether miR-129-5p could also downregulate the endogenous expression of *Fmr1* at the protein level, we assessed the FMRP expression levels by overexpressing miR-129-5p or miR-129-3p or a scrambled control in mouse N1E-115 and neural stem cells and then performed western blot analyses (Fig. 5c and data not shown). MiR-129-5p overexpression significantly reduced the expression level of FMRP, but miR-129-3p had no impact on it (Fig. 5c). Taken together, these data suggest that *Fmr1* is a direct target of miR-129-5p but not miR-129-3p.

### FMRP controls neuronal migration in the developing cerebral cortex

**Fig. 6:**
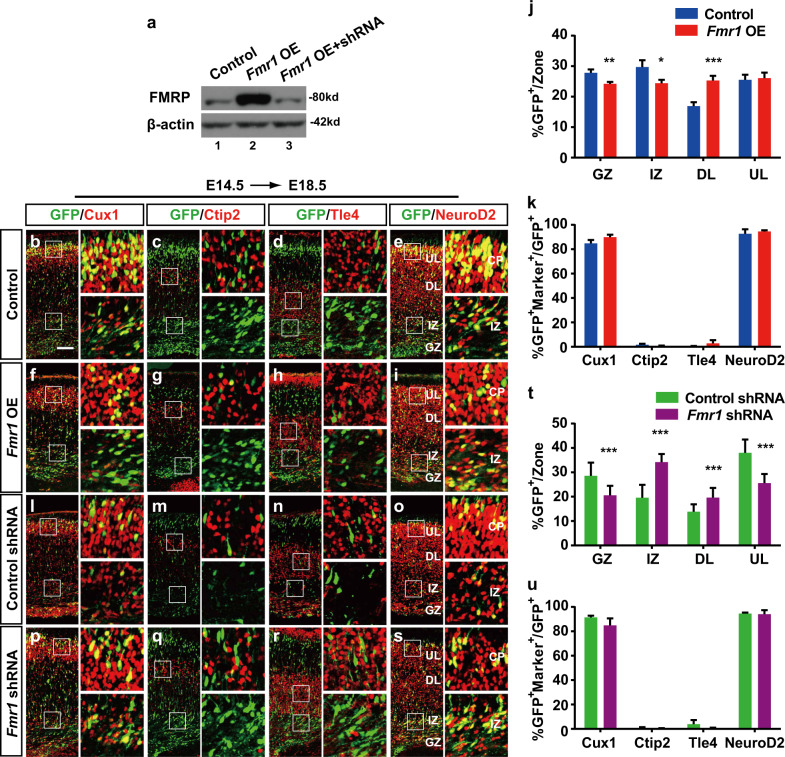
FMRP controls neuronal migration in the developing cerebral cortex. **a** *Fmr1* protein levels were detected by western blot analysis of the 293ET cells transfected after 48 h with control vectors (line 1) or *Fmr1* expression vector (line 2) or *Fmr1* expression vector together with *Fmr1* shRNA (line 3). **b**–**i**, **l**–**s** Control vector or *Fmr1* expression vector and shRNA vector containing scramble sequence (control shRNA) or *Fmr1* shRNA expression vector were electroporated into embryonic brains at E14.5, and brain sections of E18.5 embryos were stained with Cux1 (**b**, **f**, **l**, **p**), Ctip2 (**c**, **g**, **m**, **q**), Tle4 (**d**, **h**, **n**, **r**) or NeuroD2 (**e**, **i**, **o**, **s**) antibodies. Images next to each panel are high-magnification pictures of boxed regions in the CP and IZ to show the clear co-localisation of GFP^+^ cells with different layers marker. The scale bar is 100 μm in **b**. **j**, **k**, **t**, **u** Quantification of the GFP^+^ cell distribution and coexpression with the indicated markers in *Fmr1* OE (control: *n* = 3, *Fmr1* OE: *n* = 3) and KD embryos (control shRNA: *n* = 5, *Fmr1* shRNA: *n* = 3). For Ctip2 coexpressing cells, only the cells in layer V were counted. GZ germinal zone, IZ intermediate zone, DL deep layer, UL upper layer. The results are expressed as the mean ± SD or mean ± SEM. Comparisons were performed by Student’s *t*-test, and the statistically significant *P*-values are shown as *P < 0.05, **P < 001 and ***P < 0.001

Sparse data suggest that FMRP could regulate neuronal proliferation and/or differentiation as well as RGC to IP transition^42,46–48^, but its roles in neural migration remain largely unexplored. We first generated *Fmr1* OE and KD plasmids and validated their efficiency by transfecting them into 293ET cells (Fig. 6a). The expression of the *Fmr1* OE vector led to a significant upregulation of FMRP and the *Fmr1* KD plasmid abolished the increase induced by *Fmr1* OE (Fig. 6a). Next, the plasmids were transfected at E14.5, and the mice were sacrificed at E18.5. Abnormal expression of FMRP significantly increased the number of GFP^+^ cells in the deep layers at the expense of those in GZ and IZ (Fig. 6b–j), suggesting that *Fmr1* OE promotes neuronal migration. In addition, *Fmr1* OE does not affect the laminar fate of upper layer neurons (Fig. 6k).

Knockdown of FMRP at E14.5 via its shRNA significantly inhibited cortical neuronal migration, and more GFP^+^ cells were distributed in the IZ (34.2 ± 3.3% vs. 19.6 ± 5.3%) compared to those in the control embryos (Fig. 6l–t). In the E17.5 transfected neocortices, the migration defects were even more significant, and few GFP^+^ cells were present in the CP (Fig. S3). By analysing the coexpression of transfected cells outside the GZ with Cux1, Ctip2, Tle4 and NeuroD2, we found that *Fmr1* KD did not alter the cell fates of differentiated cortical neurons (Fig. 6u). These results suggest that FMRP regulates neuronal migration and has opposite phenotypes to miR-129.

### FMRP rescues the migration defect caused by miR-129

**Fig. 7:**
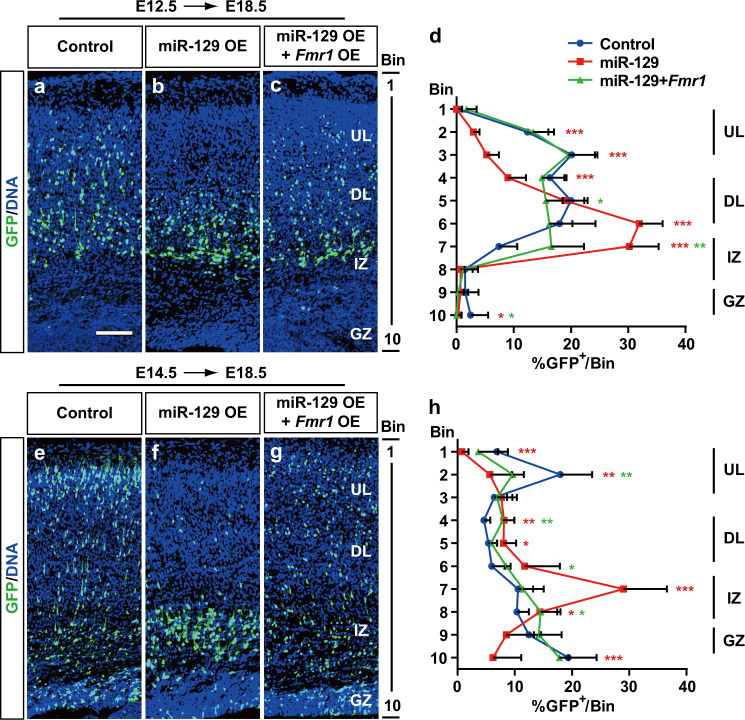
*Fmr1* rescues miR-129-repressed neuronal migration. **a**–**c**, **e**–**g** Control plasmid or plasmid expressing miR-129 (miR-129 OE) was electroporated alone or together with a *Fmr1*-expressing plasmid into embryonic mouse brains at E12.5 (**a**–**c**) or E14.5 (**e**–**g**), and brain sections of E18.5 embryos were analysed. The neocortices were divided into 10 bins for further quantification. The scale bar is 100 μm in **a**. **d**, **h** Quantification of GFP-positive cells from the sections of E18.5 embryo brains from different littermates electroporated at E12.5–E18.5 (control: *n* = 3, miR-129: *n* = 6, miR-129 + *Fmr1*: *n* = 4) and E14.5–E18.5 (control: *n* = 3, miR-129: *n* = 3, miR-129 + *Fmr1*: *n* = 3). GZ germinal zone, IZ intermediate zone, DL deep layer, UL upper layer. The results are expressed as the mean ± SD. Comparisons were performed by Student’s *t*-test, and the statistically significant *P* values are shown as *P < 0.05, **P < 001 and ***P < 0.001

To determine whether the migration defect caused by miR-129 is mediated through FMRP, we coelectroporated cells with miR-129 and FMRP expression vectors. We found that FMRP overexpression rescued the aberrant radial migration of both deep layer and upper layer neurons caused by miR-129 OE (Fig. 7a–c and e–g). Quantification of neuronal position at E18.5 confirmed that significantly more FMRP/miR-129 double-positive neurons reached the upper layers than those expressing miR-129 alone (Fig. 7d, h). Furthermore, we costained GFP^+^ cells with different layer markers to determine the identities of these cells (Fig. S4a–d, f–i). We found that FMRP and miR-129 co-overexpressing cells showed the same cell fate as control and miR-129 OE cells, both in the deep layer and upper layer neurons (Fig. S4e, j). Taken together, our results suggest that precise timing of miR-129 expression is required to fine-tune the pro-migratory function of *Fmr1*.

## Discussion

In this study, we identified a novel miRNA that regulates neuronal migration during mouse cortical development. MiR-129-5p and miR-129-3p were expressed in the NPCs and post-mitotic neurons in the CP. Ectopic expression of miR-129 resulted in the inhibition of radial migration for both the deep and upper layer neurons, and knockdown of miR-129-5p and miR-129-3p caused overmigration, suggesting that both miR-129-5p and miR-129-3p are essential for normal migration of cortical neurons. We then found that *Fmr1* was an important regulatory target for miR-129-5p. Restoring *Fmr1* expression counteracted the deleterious effect of miR-129 on neuronal migration.

Cortical neurons showed higher migratory activities from E14.5 to E16.5, during which time the expression of miR-129-5p and miR-129-3p decreased. The relatively low expression levels of miR-129-5p and miR-129-3p at a later neurogenesis time period allowed the migrating cells to reach their final position. In cancer metastasis, tumour cells with a lower level of miR-129 had stronger ability to migrate to other organs^30^. These results are consistent with our findings in cortical development, suggesting that these two systems might share common regulatory pathways. Some of the target genes for miR-129 found in human cancers could also affect neuronal migration, such as STAT3^33^, PAK5^49^ and Rab11^50^. MiR-129-3p was reported to regulate cilia biogenesis by targeting CP110 and other ciliation-related actin dynamics^51^, and defects in ciliogenesis could also cause aberrant neuronal migration, so miR-129-3p might regulate CP110 and other target genes to affect migration. We used ISH to detect miR-129-5p and miR-129-3p in transfected neocortices and found that almost all the miR-129-5p OE cells resided in the IZ, but some of the miR-129-3p OE cells migrated into the CP (Fig. 1j-n and r-v), suggesting that the inhibition of miR-129-3p on neuronal migration is weaker than that of miR-129-5p. In addition, due to the effect of miR-129-3p on migration, this may explain why FMRP only partially rescued the aberrant neuronal migration caused by abnormal miR-129 expression in our study.

FMRP, an RNA-binding protein encoded by the *Fmr1* gene, has a critical role in synaptic plasticity, dendritic morphology and processing synaptic signals^52^. The expansion of the trinucleotide CGG repeats in *Fmr1* 5′UTR results in FMRP deficiency and finally causes the Fragile X syndrome, which is a common disease belonging to the ASDs. Recent advancements in the field connected ASD to aberrant neuronal migration, and FMRP was reported to control neuronal migration in the mouse model^41^. Some studies have shown that many miRNAs, such as miR-125b, miR-132, miR-101, miR-221 and miR-130b could target *Fmr1* to regulate the molecular pathology of Fragile X syndrome via the synaptic structure and function or NPC fate determination^38,53,54^. In our study, the results indicate that miR-129-5p could negatively regulate the expression level of FMRP and is responsible for aberrant neuronal migration, and antagonising miR-129-5p may serve as a new therapeutic strategy for treating Fragile X syndrome.

## Methods

### Plasmids

Genome DNA from adult CD-1 mice was used for *pre-mir-129-2* amplification. *Pre-mir-129-2* contained a 252 bp upstream fragment, and an 84 bp downstream fragment was digested with *Xho*I and *Eco*RI and then cloned into the pCIG vector (a gift from Dr. Naihe Jing, Chinese Academy of Sciences). The complementary sequences of miR-129-5p and miR-129-3p were synthesised, annealed and inserted downstream of the luciferase gene in the pcDNA3.1 vector to construct the miR-129-5p and miR-129-3p reporter plasmids. Total complementary DNA (cDNA) from E14.5 mouse brains were used to generate the full-length 3′UTR of *Fmr1* and the open reading frame (ORF) of the *Fmr1* gene. The *Fmr1* 3′UTR was inserted downstream of the luciferase gene in the pcDNA3.1 vector. The ORF of the *Fmr1* gene was cloned into the pCIG vector. The *Fmr1* knockdown plasmid was constructed using the pLL3.7 vector (Addgene). The Fmr1-shRNA target sequence was 5′-AAGTTGAGGTTTATTCCAGAG-3′^42^, and the scramble shRNA sequence was 5′-GCGCGATAGCGCTAATAATTT-3′.

### Animals

Eight- to twelve-week-old CD-1 mice from the Beijing Vital River Laboratory Animal Limited Company (Beijing, China) were maintained in the Animal Centre of Peking Union Medical College. Animal care and experiments were approved by the Institutional Animal Care and Use Committee of the Chinese Academy of Medical Sciences and Peking Union Medical College with all procedures in compliance with the Experimental Animal Regulations (China Science and Technology Commission Order No. 2).

### In situ hybridisation (ISH)

As previously described^55^, mouse embryonic brains were fixed with 4% paraformaldehyde (PFA) in phosphate buffered saline (PBS) and then cryoprotected with 25% sucrose in PBS and equilibrated in the O.C.T. Compound (Sakura, San Diego, California, USA). Cryosections were then incubated with digoxigenin-labelled miRNA probes (Exqion, Vedbaek, Denmark) and developed following a standard ISH method. MiR-124-3p, miR-129-5p and miR-129-3p probes were purchased from Exqion.

### In utero electroporation

As previously described^23^, miRNA or *Fmr1* overexpression constructs or *Fmr1* shRNA constructs were electroporated in utero into timed pregnant CD-1 mice. Briefly, E12.5 or E14.5 pregnant dams were anaesthetised using pentobarbital sodium, and the uterine horns were exposed. Two to 3 μg/μl of plasmids or 40 µM miRNA antagomirs spiked with Fast Green (Sigma, Louis, Missouri, USA) were injected into the lateral ventricle of the embryo brain. Electroporation was conducted with electric pulses of 20–30 V for 50 ms, which were repeated five times with 950-ms intervals using the BTX-ECM830 electroporator (Harvard Apparatus, Holliston, Massachusetts, USA).

### Immunohistochemistry

Immunohistochemical analyses of the brain cryosections were conducted as previously described^23^. Mounting medium with DAPI (F6057, Sigma) was used for DNA staining and mounted with a coverslip. The primary antibodies used for IHC were as follows: pHH3 (Abcam, Cambridge, UK), Pax6 (Convance, Princeton, New Jersey, USA), Tbr2 (Abcam), Cux1 (Santa Cruz Biotechnology, Dallas, Texas, USA), Ctip2 (Abcam), Tle4 (Santa Cruz Biotechnology), NeuroD2 (Abcam), and S100 (Abcam). Secondary antibodies were Alexa Fluor 594 (Invitrogen, Waltham, Massachusetts, USA). EdU staining was performed using a Click-iT™ EdU Imaging Kit (Invitrogen) according to the manufacturer’s instructions. Images were collected using an Olympus F1000 Confocal Microscope and processed using FV10-ASW 3.0 Viewer and Adobe Photoshop.

### Cell culture

The HEK-293ET cells (kindly provided by Dr. Chengyu Jiang, Peking Union Medical College) and N1E-115 cells (kindly provided by Dr. Yan Zhou, Wuhan University) were cultured in complete Dulbecco’s modified Eagle’s medium (DMEM) (Thermo Scientific, Waltham, Massachusetts, USA) with 10% (v/v) foetal bovine serum (FBS). For transfections, plasmids, miRNA mimics (GenePharma, Shanghai, China) and miRNA antagomirs (RiboBio, Guangzhou, China) were transfected into HEK-293ET cells using Lipofectamine 2000 (Invitrogen) according to the appropriate manufacturer’s instructions.

### Dual-Luciferase reporter assay

As previously described^56^, the 3′ UTR of *Fmr1* was amplified from mouse cDNA and inserted downstream of the firefly luciferase gene. Cells were seeded in 24-well plates and transfected with luciferase reporters (pRL-TK, 50 ng/well; Firefly luciferase reporter, 200 ng/well) and miRNA mimics (25 nM) after 48 h. Forty-eight hours later, the cells were lysed, and the luciferase activity was determined using a Dual Luciferase Reporter Assay System (Promega, Fitchburg, Wisconsin, USA). MiRNA mimics were purchased from the Shanghai GenePharma Company. MiRNA antagomirs were purchased from the Guangzhou RiboBio Company.

### Western blot analysis

Total protein was extracted from cultured cells using protein lysis buffer (50 mmol/L Tris, pH 7.5, 150 mmol/L NaCl, 2 mmol/L EDTA and 1% Triton X-100) supplemented with protease inhibitors (Roche, Rotkreuz, Switzerland). Protein samples were separated on a SDS- polyacrylamide gel electrophoresis (PAGE) gel followed by western blotting using FMRP antibody (F4055, Sigma) and β-actin antibody (A5441, Sigma).

### Statistical analysis

The results are expressed as the mean ± standard deviation. Statistical analyses were performed by Student’s *t*-test using GraphPad Prism 6, with *P*-values < 0.05 considered statistically significant.

## Supplementary information

Supplemental Information-miR-129 and migration
