# Supplementary material for: MicroRNA-129 modulates neuronal migration by targeting Fmr1 in the developing mouse cortex
Source: Cell Death Dis. 2019 Mar 25;10(4):287. doi: 10.1038/s41419-019-1517-1 (PMC6433925; doi:10.1038/s41419-019-1517-1)
Supplement: Supplementary file 1 — Supplemental Information-miR-129 and migration [file 41419_2019_1517_MOESM1_ESM.doc]

**MicroRNA-129** **modulates** **neuronal migration** **by**

**targeting *Fmr1* in the developing mouse cortex**

**Chao Wu^1^****^†^, Xiaoling Zhang^1†^, Pan Chen^1^, Xiangbin Ruan^1^, Wei Liu^3^, Yanchao Li^4^, Changjie Sun^1^, Lin Hou^1^, Bin Yin^1^, Boqin Qiang^1^, Pengcheng Shu^1^* and Xiaozhong Peng^1,2^***

**Running Title:** MicroRNA-129 modulates neuronal migration

**Supplemental Information:**

Includes 4 figures.

**
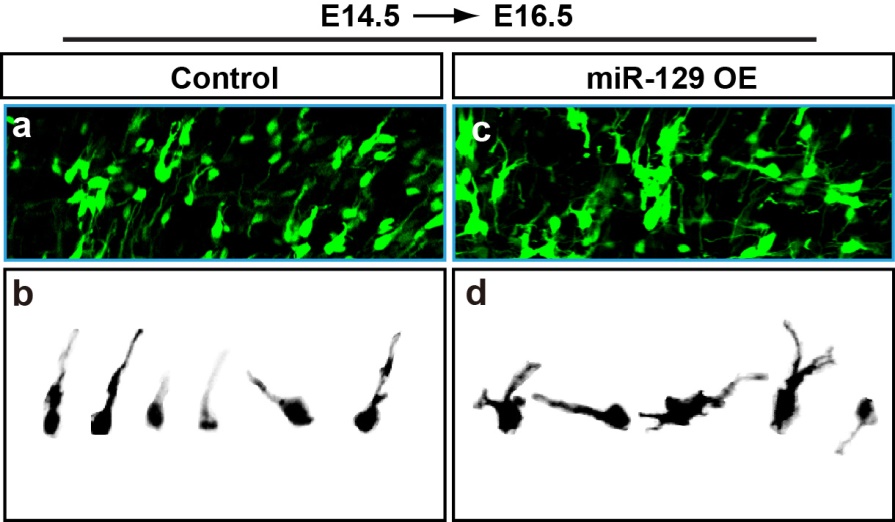
**

**Figure S1. MiR-129 regulates the multipolar to bipolar transition** **affecting neuronal migration**

(a-d) The coronal sections were transfected with control and miR-129 OE vectors at E14.5 and analysed at E16.5 to examine the morphology of migrating neurons.

**
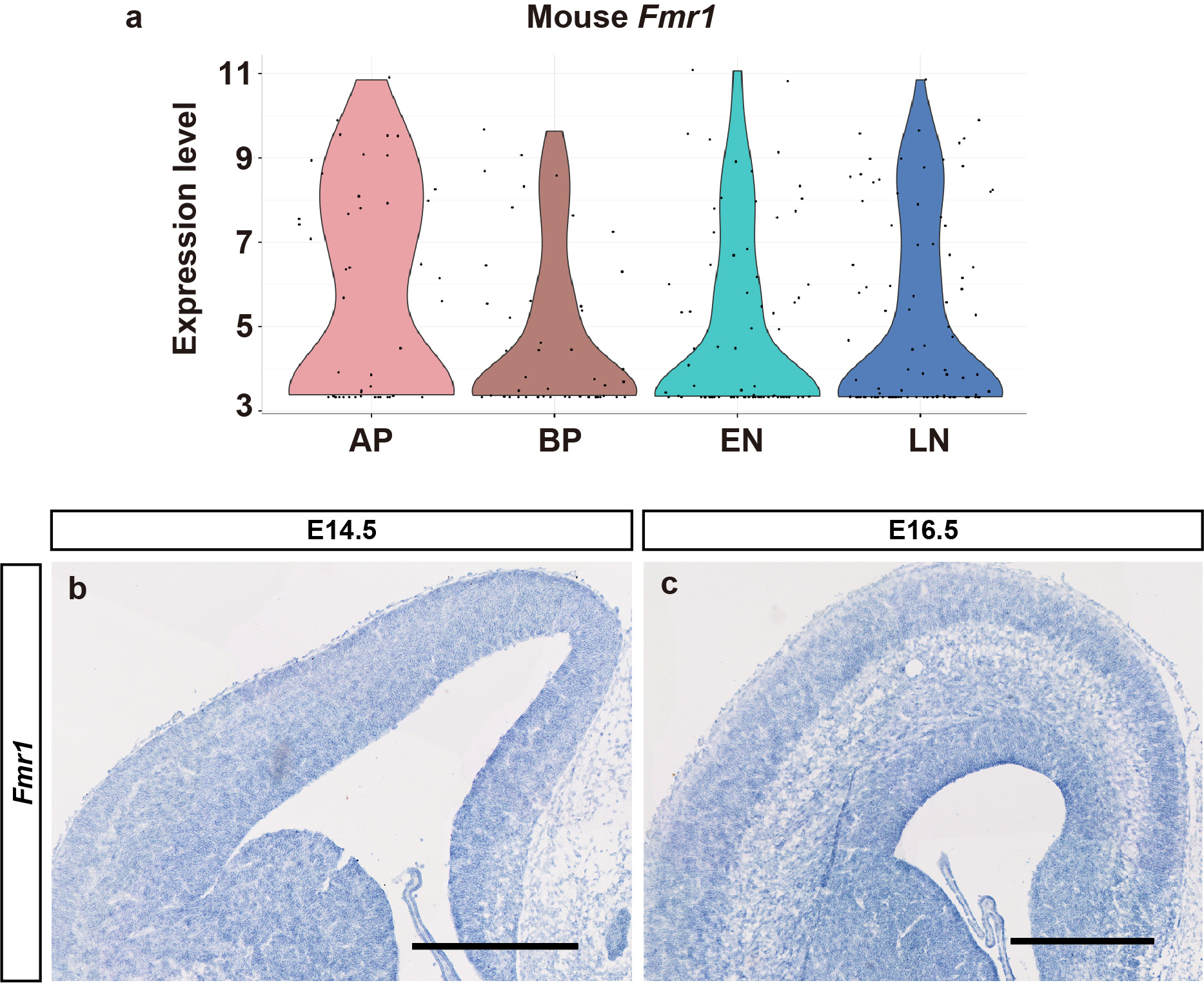
**

**Figure S2. The** **expression patterns of Fmr1 in the developing mouse cortex**

(a) Single-cell transcriptomics in the Science paper published in 2016. The expression pattern of *Fmr1* in different cell types. AP: Apical progenitors; BP: daughter basal progenitors; EN: Early neurons; LN: Late neurons. (b and c) The expression patterns of *Fmr1* in the mouse dorsal forebrain at E14.5 and E16.5. The scale bar is 500μm in b and c.


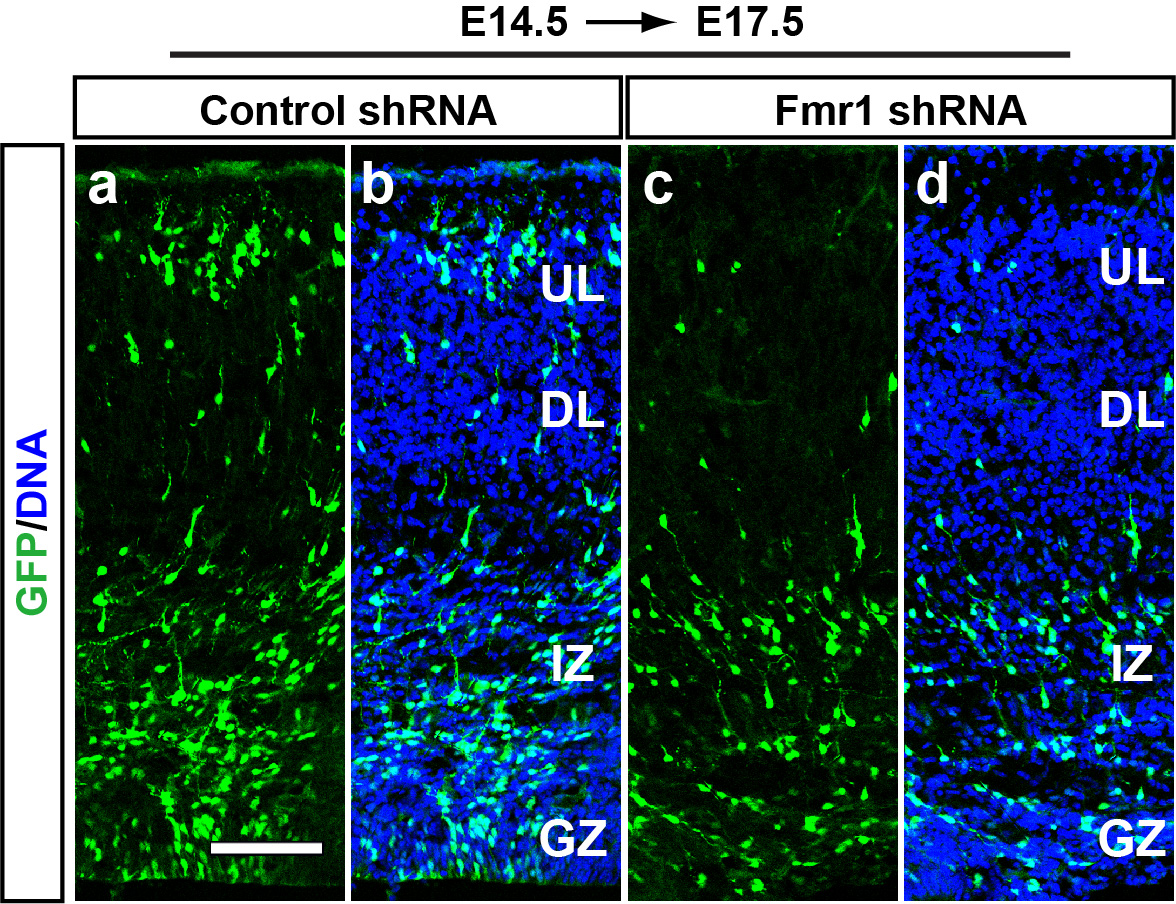


**Figure S3. FMRP regulates neuronal migration in the developing mouse neocortex**

(a-d) Control scramble shRNA vector (control shRNA) or *Fmr1* shRNA expression vector (Fmr1 shRNA) were electroporated into embryonic brains at E14.5, and brain sections of E17.5 embryos were analysed using DAPI and confocal microscopy. The scale bar is 100μm in a. GZ, germinal zone. IZ, intermediate zone. DL, deep layer. UL, upper layer.


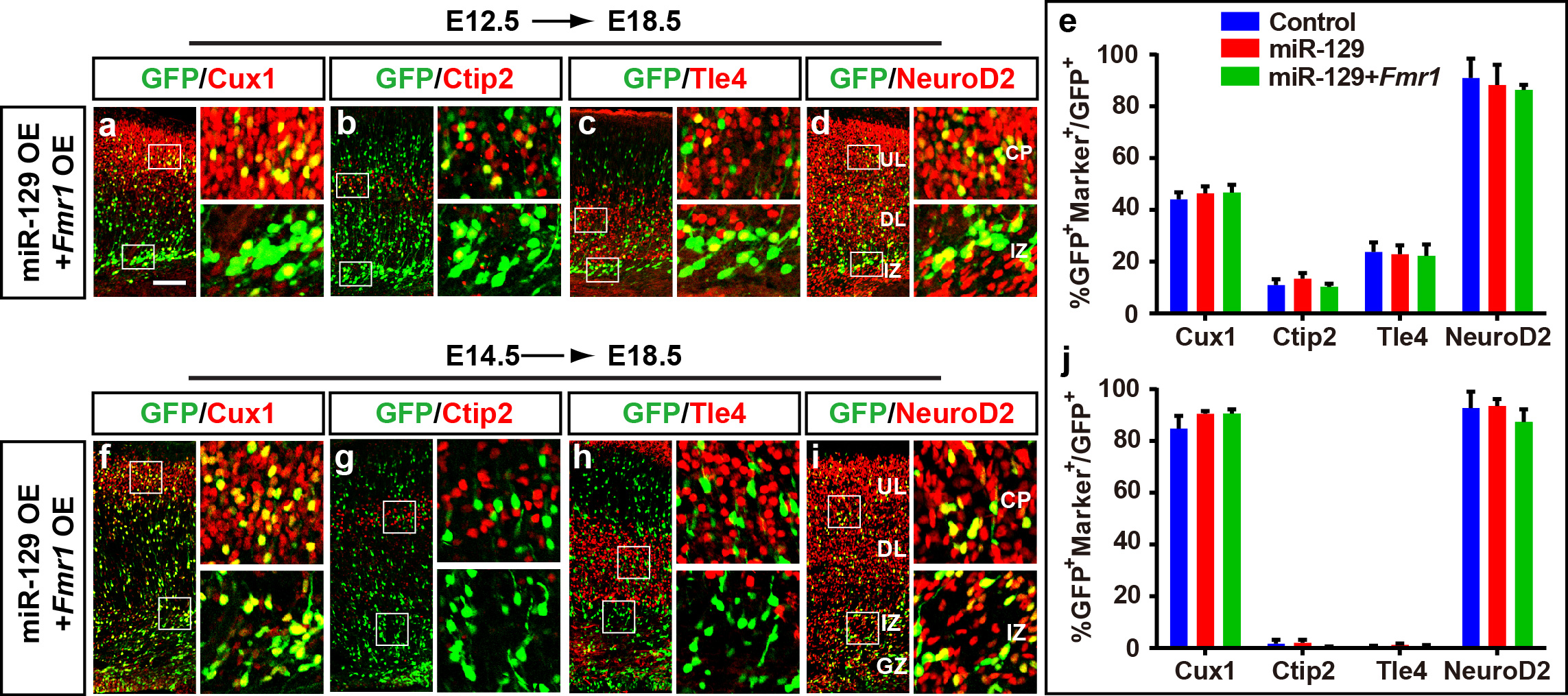


**Figure S4. MiR-129 and FMRP-coexpressing cells show the same cell fates as control and miR-129-overexpressing cells during neurogenesis.**

(a-d and f-i) MiR-129 expression plasmids and *Fmr1* expression plasmids were electroporated into the embryonic brains at E12.5 (a-d) or E14.5 (f-i), and brain sections of E18.5 embryos were stained with Cux1 (a and f), Ctip2 (b and g), Tle4 (c and h) or NeuroD2 (d and i) antibodies. Images next to each panel are high-magnification pictures of boxed regions in the CP and IZ to show the clear colocalization of GFP^+^ cells with different layer markers. The scale bar is 100 μm in a. (e and j) GFP^+^ cells coexpressing the indicated markers were quantified from different littermates with E12.5-E18.5 electroporations (control: n=3, miR-129: n=6, miR-129+Fmr1: n=4) and E14.5-E18.5 electroporations (control: n=3, miR-129: n=3, miR-129+Fmr1: n=3). For Ctip2 coexpressing cells, only the cells in layer V were counted. GZ, germinal zone. IZ, intermediate zone. DL, deep layer. UL, upper layer. The results are expressed as the mean±SD. Comparisons were performed by Student’s t test, and the statistically significant P values are shown as *P<0.05, ** P<0.01 and ***P<0.001.
